# Hypovitaminosis D in Postherpetic Neuralgia—High Prevalence and Inverse Association with Pain: A Retrospective Study

**DOI:** 10.3390/nu11112787

**Published:** 2019-11-15

**Authors:** Jen-Yin Chen, Yao-Tsung Lin, Li-Kai Wang, Kuo-Chuan Hung, Kuo-Mao Lan, Chung-Han Ho, Chia-Yu Chang

**Affiliations:** 1 Department of Anesthesiology, Chi Mei Medical Center, Tainan 71004, Taiwananesth@gmail.com (L.-K.W.); ed102605@gmail.com (K.-C.H.); albklan@gmail.com (K.-M.L.); 2 Department of the Senior Citizen Service Management, Chia Nan University of Pharmacy and Science, Tainan 71004, Taiwan; 3 Department of Medical Research, Chi Mei Medical Center, Tainan 71004, Taiwan; ho.c.hank@gmail.com; 4 Department of neurology, Chi Mei Medical Center, Tainan 71004, Taiwan; chiayu.chang7@msa.hinet.net; 5 The center for General Education, Southern Taiwan University of Science and Technology, Tainan 71004, Taiwan

**Keywords:** hypovitaminosis D, 25-hydroxyvitamin D, postherpetic neuralgia, spontaneous pain, brush-evoked pain, varicella-zoster virus immunoglobulin, DN4questionnaire

## Abstract

Hypovitaminosis D (25-hydroxyvitamin D (25(OH)D) <75 nmol/L) is associated with neuropathic pain and varicella-zoster virus (VZV) immunity. A two-part retrospective hospital-based study was conducted. Part I (a case-control study): To investigate the prevalence and risk of hypovitaminosis D in postherpetic neuralgia (PHN) patients compared to those in gender/index-month/age-auto matched controls who underwent health examinations. Patients aged ≥50 years were automatically selected by ICD-9 codes for shingle/PHN. Charts were reviewed. Part II (a cross-sectional study): To determine associations between 25(OH)D, VZV IgG/M, pain and items in the DN4 questionnaire at the first pain clinic visit of patients. Independent predictors of PHN were presented as adjusted odds ratios(AOR) and 95% confidence intervals (CI). Prevalence (73.9%) of hypovitaminosis D in 88 patients was high. In conditional logistic regressions, independent predictors for PHN were hypovitaminosis D (AOR3.12, 95% CI1.73–5.61), malignancy (AOR3.21, 95% CI 1.38–7.48) and *Helicobacter pylori*-related peptic ulcer disease (AOR3.47, 95% CI 1.71–7.03). 25(OH)D was inversely correlated to spontaneous/brush-evoked pain. Spontaneous pain was positively correlated to VZV IgM. Based on the receiver operator characteristic curve, cutoffs for 25(OH)D to predict spontaneous and brush-evoked pain were 67.0 and 169.0 nmol/L, respectively. A prospective, longitudinal study is needed to elucidate the findings.

## 1. Introduction

Vitamin D is essential for musculoskeletal health in humans. The major circulating form of vitamin D is serum 25-hydroxyvitamin D (25(OH)D)—the main storage form. Currently, serum total 25(OH)D is considered to be the best marker of vitamin D status among the possible markers [1]. However, the definition of hypovitaminosis D is a central controversy in vitamin D research [1]. In the present study, sufficiency of vitamin D is defined as 25(OH)D ≥ 75 nmol/L (30 ng/mL) as defined by the Endocrine Society Clinical Practice Guideline [2]. Low vitamin D (hypovitaminosis D) includes insufficiency (50–75 nmol/L) and deficiency (< 50 nmol/L, 20 ng/mL) [2,3]. This cut-off is based on studies showing an increased intestinal calcium absorption and a decreased level of circulating parathyroid hormone when 25(OH)D levels were >75 nmol/L [2]. Notably, extra-skeletal functions of vitamin D are increasingly recognized. Vitamin D possesses anti-viral effects through vitamin D-induced peptides [4,5]. Vitamin D can inhibit neuroinflammation by downregulation of proinflammatory cytokines and upregulation of anti-inflammatory cytokines [6,7]. Vitamin D has concentration-dependent anti-inflammatory effects on glia and astrocytes through inhibiting the production of nitric oxide (NO) [8,9]. Importantly, NO increases phosphorylated N-methyl-D-aspartate (NMDA) receptors in spinal dorsal horn neurons which have been shown to be essential for the initiation of central sensitization and the development of mechanical allodynia [10]. In animal neuropathic pain models, vitamin D deficiency increases the production of reactive oxygen species (ROS) [11] which result in cold pain via the activation of transient receptor potential ankyrin 1 (TRPA1) [12,13] and contribute to mechanical hyperalgesia via the enhancement of NMDA receptor activation [14,15]. Vitamin D deficiency induces a marked dysbiosis and alters nociception possibly via molecular mechanisms involving the endocannabinoid and related mediator signaling system [16]. Vitamin D supplementation reduces mechanical hyperalgesia and cold allodynia [17]. Hypovitaminosis D has been demonstrated to increase neuropathic pain in patients with diabetic and rheumatoid arthritis [18,19,20]. Overall, vitamin D deficiency is associated with increased neuropathic pain.

Herpes zoster (shingles) is a common infectious disease resulting from reactivation of latent varicella-zoster virus (VZV). Patients with shingles suffer from herpetic pain which generally subsides within four weeks. Approximately 8%–24% of all zoster patients develop chronic herpetic pain known as postherpetic neuralgia (PHN) which lasts longer than 90 days after rash onset [21,22,23]. Although shingles vaccines are effective for preventing shingles and PHN, one-third of vaccinated persons aged ≥60 still develop PHN [24]. Besides, some PHN patients show inadequate responses to current therapies [25]. Thus far, PHN treatment remains challenging. Chronic pain in older patients may impair their ability to perform activities of daily living [26], leading to the development of vitamin D deficiency. Serum 25(OH)D status is positively associated with zoster immunity in dialysis patients [27]. We, therefore, conducted a two-part retrospective hospital-based study. In Part (a case-control study), we investigated the prevalence and the risk of hypovitaminosis D in PHN patients compared to those in controls receiving health examinations.

PHN is a peripheral neuropathy PHN patients experience various spontaneous and/or brush-evoked pain (allodynia) [28]. The Douleur Neuropathique 4 (DN4) questionnaire, which consists of seven symptoms (burning, painful cold, electric shocks, tingling, pins and needles, numbness, itching) and three items of physical examination (hypoesthesia to touch and pinprick as well as brush-evoked pain), is a popular tool for assessing the probability of neuropathic pain [29]. Following the protocol of our previous studies and that of others [28,30,31,32,33], zoster patients in the current study routinely received nutrient survey (e.g., serum 25(OH)D) and completed questionnaires including the DN4 questionnaire and those on pain intensity during their first visits to our pain clinic (Supplementary S1). Subsequently, the patients recruited for case-control study (i.e., Part I) were enrolled in Part II study (i.e., a cross-sectional study) that assessed the associations among vitamin D status, zoster immunity (VZV immunoglobulins), spontaneous/brush-evoked pain, and 10 items in the DN4 questionnaire [27,30,34,35] in PHN patients.

## 2. Materials and Methods 

The study was conducted in accordance with the Declaration of Helsinki. This retrospective study was approved by the Institutional Review Board of the Chi Mei Medical Center in Tainan, Taiwan (IRB-10606-002). Patients’ data in this study were drawn from the electronic medical database of Chi Mei Medical Center which is a 1200-bed tertiary referral center in Tainan, Taiwan.

During their first pain clinic visit, zoster patients routinely completed the DN4 questionnaires (seven questions and three clinical examinations) and received evaluation of their average daily spontaneous pain severity and the worst spontaneous pain on an 11-point numeric rating pain scale (NRS, 0: no pain; 10: worst pain imaginable) [28] (Supplementary S1). Brush-evoked pain (mechanical allodynia) was assessed by using a manual handheld cotton swab swept three times approximately 3–5 cm in length over the skin, with a speed of 1 cm/s. A positive test of brush-evoked pain was defined as pain sensation elicited by at least two out of three strokes. The intensity of brush-evoked pain was graded on the 11-point NRS (0–10). Fasting blood samples were collected in the morning after the patients’ pain clinic visits for 25(OH)D and VZV IgG/IgM antibody tests. In our pain clinic, a serum 25(OH)D survey has been performed routinely in zoster patients since 2011 [28,30,31,36]. Additionally, serological tests for VZV IgG/IgM have been a routine for the differential diagnosis of shingles from other skin diseases in zoster patients since mid-2012 [27,30,31,34,35]. Patients’ information and data were collected and recorded in the electronic medical database.

### 2.1. Autosearch and Chart Review Criteria for Postherpetic Neuralgia

In this retrospective study, zoster/PHN patients aged ≥ 50 [37] receiving serum 25(OH)D and VZV IgG/IgM survey were selected by auto matched search from the computerized database (June 1, 2012–Dec. 31, 2016). Inclusion criteria for PHN were (1) International Classification of Diseases, Ninth Revision, Clinical Modification codes (ICD-9) 053 herpes zoster/053.X combined with new prescriptions of an analgesic, an anticonvulsant or an antidepressant for at least 90 days; (2) ICD-9 053.1X (herpes zoster with nervous system complications) [37,38]; and (3) patients with pain clinic visits ≥ 2 times during the study period [37,38]. Exclusion criteria were as follows: (1) patients diagnosed with herpes zoster ICD-9 053 in the preceding years; (2) patients who had diagnostic codes of human immunodeficiency virus infection (ICD-9 042, 043, 044) and organ transplants (ICD-9 3751, 1160, 1164, 1169, 5059, 5280, 5283, 5569, 3350–3352), which are potential confounders of shingles/PHN [21,38] and hypovitaminosis D [39,40]; and (3) patients whose medical records showed no evidence of serum 25(OH)D and VZV IgG/IgM survey during the study period.

Physicians (JYC and YTL) signed a patient confidentiality agreement before chart reviews. Each chart was reviewed for the inclusion and exclusion criteria. We included PHN patients who received a prescribed analgesic, anticonvulsant, or antidepressant or treatments by physicians for persisting pain ≥ 3 months and shorter than two years after zoster rash onset [28] as well as having a worst pain score ≥ 4 on the 11-point NRS [25]. In addition, no other cause for pain was considered more likely than PHN.

**Figure 1:**
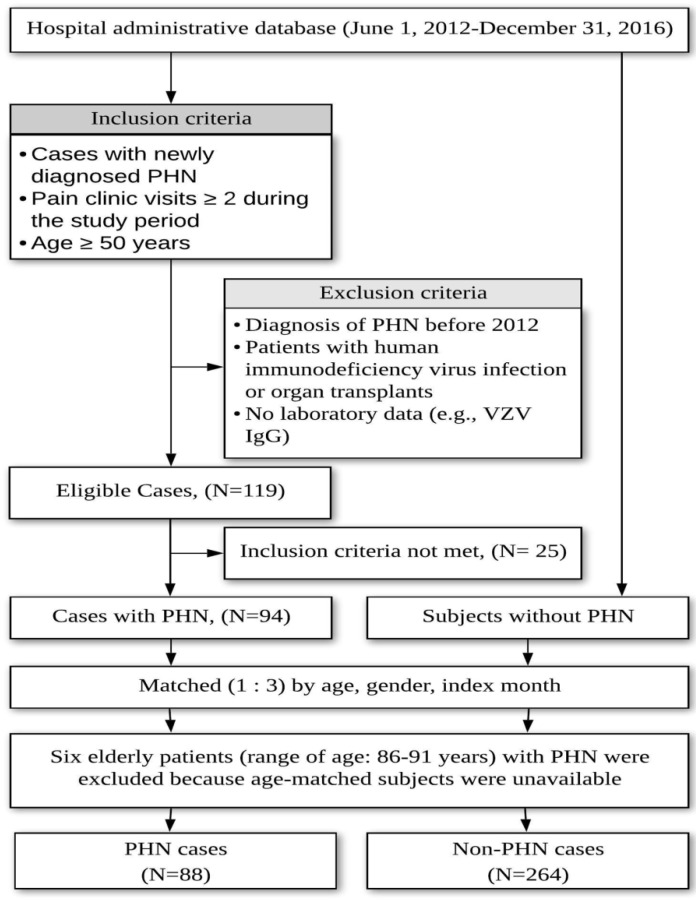
Flowchart of participant recruitment and case–control selection in Part I study.

Age, gender and index-month are common confounders of vitamin D status [41]. Conditional logistic regression models were used by matching gender, index month, and age (i.e., ≤ 2 years between the two groups). The auto matched controls were individuals who received a health examination survey during the same period when the study patients visited the pain clinic. The ratio of controls to patients was 3 to 1 (Figure 1). The health survey package of our hospital included routine gastroduodenoscopy and 25(OH)D quantification [42].

The present study was a two-part retrospective hospital-based study including Part I (a case-control study) and Part II (a cross-sectional study). Using data from the electronic medical database of our hospital, we included automatically selected PHN patients and the gender/index-month/age-auto matched controls (non-PHN cases) in the case-control study. In the cross-sectional analysis, we included participants who were PHN patients of the case-control study.

### 2.2. Comorbidities

Hypertension/diabetes mellitus diagnosis was defined as patients who had ICD-9 codes for hypertension (ICD-9 401-405)/diabetes mellitus (ICD-9 250) and received a prescribed medication for hypertension/diabetes mellitus. Malignancy, autoimmune diseases, and chronic liver disease diagnosis were defined as patients who had ICD-9 code (malignancy 140-239; autoimmune diseases 710, 714, 725, 555, 556, 696, 340, 245.2; chronic liver disease 571) and received treatments by physicians for the diseases. Patients with chronic kidney disease were those who had ICD-9 585 and received dialysis regularly. *Helicobacter pylori*-related peptic ulcer disease was identified by ICD-9 530–534 and confirmed by positive findings on either hospital gastroduodenoscopy records or a self-reported gastroduodenoscopy history with prescriptions for the disease within one year prior to shingles outbreak [38].

### 2.3. Specimen Collection, Handling, and Biochemical Determination

#### 2.3.1. Determination of 25(OH)D

Automated immunoassays are currently popular methods for measuring the circulating level of 25(OH)D. At our institute, a fasting blood sample was drawn in the morning from the testing subjects. Once the serum was separated, it was kept frozen at a temperature of −70℃ until analysis. Serum 25(OH)D concentrations were measured by ARCHITECTi2000 (Abbott, Chicago, IL, USA) (Chemiluminescent Microparticle Immuno Assay) [43] every weekday.

#### 2.3.2. Zoster Immunity—VZV IgG/IgM Detected by ELISA

Serum samples of PHN patients were routinely obtained for the VZV IgG and IgM antibody tests using enzyme-linked immunosorbent assay (ELISA) kits manufactured by Euroimmun (Lübeck, Germany) [44] and TECAN washer. The absorbance [44] was measured using ELISA reader Multiskan FC Thermo Scientific (Waltham, MA, USA). The result for VZV IgG was determined to be positive with the cut-off  ≥  110 mIU/mL. A positive result of VZV IgG was considered good immunity against VZV [44]. Positivity for VZV IgM was defined as an antibody index ≥ 1.0 [45]. A positive IgM result indicates recent or current VZV infection [27,34]. All assays were performed at the Chi Mei Medical Center Laboratory according to the instructions of the manufacturer. 

### 2.4. Sample Size

In this study, the estimated rate of hypovitaminosis D in patients versus the controls was 70% versus 50% [3]. Therefore, a minimum sample size of patients (84 in each group) was determined to ensure a high power with a 5% significance level for an analysis of the difference between PHN patients and the controls.

### 2.5. Statistical Analysis 

Data processing and statistical analysis were performed using SAS statistical software (Version 9.4; SAS Institute, Cary, NC, USA). The significance of difference among continuous data between the two groups was determined by student’s t-test. Chi-square test or Fisher exact test was used to test the differences in categorical variables between the two groups. The risk of PHN was presented as an odds ratio (OR) and 95% confidence intervals (CI). Patients were divided into two groups according to age: patients aged 60years or older (*n* = 64) and patients aged 50–59 years (*n* = 24). Univariate logistic regression analysis was used to examine the associations between all selected predictors and PHN development in this study. A univariate association (*p* < 0.10) with PHN was included in the conditional multiple logistic regression model. Independent predictors for PHN were identified in the conditional multiple logistic regression model by gender, index month, and age (i.e., ≤ 2 years between two groups) match. Furthermore, patients were divided into two groups according to 25(OH)D levels: hypovitaminosis D (25(OH)D < 75 nmol/L) and sufficiency of vitamin D (25(OH)D ≥ 75 nmol/L). All of the demographic and clinical variables were compared between patients with sufficient-vitamin D and those with hypovitaminosis D.

The normality of variables was examined with the Kolmogorov–Smirnov test. Pearson’s or Spearman’s correlation was performed to test the significance of the association between clinical variables (e.g., 25(OH)D, VZV Ig) and severity of pain where appropriate. The correlation between clinical variables and severity of pain was considered to be clinically significant if the rho>0.3 [28]. According to pain severity, PHN patients were dichotomized into two pain groups: patients with mild pain (NRS ≤ 5) and those with moderate to severe pain (NRS 6–10). For identifying the optimal cutoff point for these clinical variables (e.g., 25(OH)D, VZV Ig) in predicting moderate to severe pain (i.e., NRS 6–10), a receiver operating characteristic (ROC) curve was plotted. The optimal cutoff value was determined with the Youden’s index via maximizing the point on the ROC curve furthest from the line of equality. The area under the ROC curve (AUC) was used to measure the diagnostic ability of a variable (e.g., 25(OH)D, VZV Ig). Furthermore, the proportions of items in the DN4 questionnaire between patients with 25(OH)D /VZV IgM ≤ the cutoff point and those with levels > the cutoff point were compared to identify the associations between 25(OH)D /VZV IgM and symptoms/physical findings. A *p* value of <0.05 was considered statistically significant.

## 3. Results

A total of 119 PHN medical records were selected for review. Three patients were considered to experience other causes of chronic pain, while 19 patients were determined to suffer from zoster-associated pain which was defined as herpetic pain beyond 30 days but less than 90 days. Three patients were excluded due to incomplete records. In total, 25 patients were excluded after medical record review. Additionally, six elderly patients were excluded because of no age-matched controls (Figure 1).

### 3.1. Part I Study

Conditional Logistic Analysis for the Predictors of Postherpetic Neuralgia

The demographic characteristics of 88 patients and 264 controls are shown in Table 1. Comparisons between patients and the controls showed that PHN patients had significantly lower serum 25(OH)D (68.96 nmol/L, SD 18.72 nmol/L) and higher prevalence of hypovitaminosis D (73.9%) than those (75.13 nmol/L, SD17.47nmol/L; 47.0%) in the controls (*p* = 0.005; <0.001). Furthermore, PHN patients had higher prevalence of diabetes mellitus (29.5% vs. 15.9%, *p* = 0.005), malignancy (17.0% vs. 6.8%, *p* = 0.007) and *Helicobacter pylori*-related peptic ulcer disease (26.1% vs. 9.5%, *p* < 0.001) compared to that in the controls. There were no significant differences inbody mass index and the prevalence of hypertension, autoimmune diseases, chronic liver and kidney disease between the two groups.

Although four risk factors for PHN were identified in univariate conditional logistic analysis, only three risk factors remained after conditional multivariate logistic analysis, including hypovitaminosis D (adjusted OR: 3.12, 95% confidence interval (CI) 1.73–5.61, *p* < 0.001), malignancy (adjusted OR: 3.21, 95% CI 1.38–7.48, *p* = 0.007) and *Helicobacter pylori*-related peptic ulcer disease (adjusted OR: 3.47, 95% CI 1.71–7.03, *p* = 0.001).

### 3.2. Part II Study

#### 3.2.1. Comparison of Demographic and Clinical Characteristics Between Vitamin D-Deficient Patients and Vitamin D-Sufficient Patients

Patients with hypovitaminosis D had higher VZV IgM titers (0.63, SD 0.45), a lower vitamin D supplementation rate (1.5%) and greater spontaneous/brush-evoked pain intensity (6.1, SD 2.1; 4.3, SD 6.8), compared to those in patients with sufficient vitamin D (0.40, SD 0.25; 17.4%; 5.3, SD 1.8; 2.5, SD 8.3) (*p* = 0.016; 0.005; 0.021; 0.007) (Table 2).

#### 3.2.2. Correlations Between Pain and Serum 25(OH)D/VZV Igs in PHN

In Table 3, spontaneous pain was correlated to serum concentrations of 25(OH)D (Spearman correlation coefficient: −0.329, *p* = 0.002) and VZV IgM (Spearman correlation coefficient: 0.363, *p* = 0.001). Brush-evoked pain was correlated to the serum level of 25(OH)D (Spearman correlation coefficient: −0.311, *p* = 0.003). The other Spearman correlation coefficients were ≤ 0.3 indicating no clinical significance (Data not shown).

**Figure 2:**
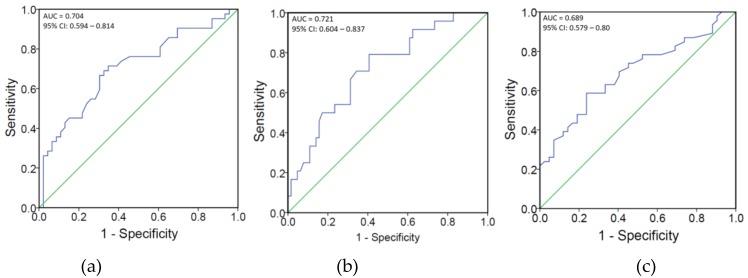
(**a**)area under the receiver operating characteristic curve for 25(OH)D concentration in spontaneous pain; (**b**)the area under the receiver operating characteristic curve for 25(OH)D status in brush-evoked pain; (**c**)the area under the receiver operating characteristic curve for IgM titer in spontaneous pain.

The cutoffs for serum 25(OH)D concentration to predict spontaneous pain and brush-evoked pain were 67.0 nmol/L (26.8 ng/mL) (sensitivity 71.4%; specificity 65.2%) and 169.0 nmol/L (67.6 ng/mL) (sensitivity 79.2%; specificity 59.4%), respectively. The cutoff for IgM titer to predict spontaneous pain was 0.60 with a sensitivity of 58.7% and a specificity of 76.2%. Based on the AUC values, we found that serum 25(OH)D status (0.704; 0.721) and IgM titers (0.689) were good predictors for pain in PHN (Figure 2a–c).

#### 3.2.3. Proportions of 10 Items in the DN4 Questionnaire in Patients with Different Serum 25(OH)D/VZV Igs

In 88 PHN patients, 74 patients (84.1%) had a score greater or equal to 4 in the DN4 questionnaire. In10items of the DN4 questionnaire, patients with vitamin D ≤ 67.0 nmol/L had greater proportions of painful cold and brush-evoked pain compared to those in patients with vitamin D > 67.0 nmol/L (*p* < 0.001; *p* = 0.002). Although vitamin D-deficient patients (< 50.0 nmol/L) had greater proportions of painful cold and brush-evoked pain compared to those in vitamin D-insufficient patients (*p* = 0.005; *p* = 0.225), no statistically significant difference in brush-evoked pain was found between the two groups. Possibly, it was due to the small case number in the group with deficiency. (Table 4)

For the10 items in the DN4 questionnaire, no significant finding was noted in patients with IgM ≤ 0.6 compared to those with IgM > 0.6. (Table 5).

## 4. Discussion

The current study demonstrated a significantly higher prevalence of hypovitaminosis D in PHN patients than that in the controls. The rate in the controls was similar to that (44.1%) in subjects living on similar latitudes in our country located in the subtropical region [3]. The present study also showed that, compared to vitamin D-sufficient subjects, PHN patients with hypovitaminosis D had a lower vitamin D supplementation rate, greater pain intensity, and higher VZV IgM titers. There were several possible explanations for the high prevalence of hypovitaminosis D among PHN patients. First, compared to the healthy controls, PHN patients had a higher prevalence of diabetes mellitus [18], malignancy [46] and *Helicobacter pylori*-related peptic ulcer disease [42], all of which have been linked to hypovitaminosis D. Second, because low vitamin D intake is a known independent predictor of hypovitaminosis D [3], lack of vitamin D supplementation in the majority (94.3%) of patients may contribute to this condition. Third, previous studies have reported elevated titers of VZV IgM titers in patients with hypovitaminosis D [27,45], indicating a current virus infection. Because vitamin D could enhance antimicrobial peptide expression [4,5], hypovitaminosis D may suppress antimicrobial peptide production and facilitate chronic infection. Moreover, higher VZV IgM titers in patients with hypovitaminosis D imply persistent VZV infection that may contribute to chronic pain and its severity. Chronic pain of high severity, in turn, could cause impaired activities of daily living in patients [26], leading to vitamin D deficiency. Interestingly, a previous report showed a high prevalence of hypovitaminosis D in hospitalized patients with shingles [47]. It raised a question of whether hypovitaminosis D in PHN was pre-existing at the onset of shingles or it was the consequence of depletion from PHN-related pain or chronic viral infection [27,30,35,47]. Instead of identifying causality, a retrospective case-control study could only establish an association. Longitudinal and experimental research is needed to further elucidate the findings.

In Part I study, hypovitaminosis D, malignancy and *Helicobacter pylori*-related peptic ulcer disease independently predicted PHN in conditional multiple logistic analysis. Because the pathogenesis of PHN includes neuronal excitability and persistent viral infection-induced neuroinflammation [48,49], there are probable molecular associations between hypovitaminosis D and PHN. First, activated microglia and astrocytes have been found to cause neuronal excitability, leading to neuropathic pain [49]. On the other hand, vitamin D can inhibit the activation of microglia [50] and astrocytes [51]. Second, vitamin D inhibits neuroinflammation by suppressing the production of pro-inflammatory cytokines and increasing that of anti-inflammatory cytokines [7,52]. Third, vitamin D possesses a direct anti-viral effect by enhancing the expression of antimicrobial peptides to suppress VZV replication in keratinocytes and B cells [4,5]. Taken together, hypovitaminosis D may induce PHN as a result of hyper-excitability of neurons, neuroinflammation and persistent viral replication. 

PHN patients experience various spontaneous pain and brush-evoked pain (allodynia) [28]. Significant inverse associations were found between serum 25(OH)D level and the presence of spontaneous/brush-evoked pain in PHN patients. Spontaneous pain is related to the neuroinflammation-induced spontaneous firing of intact C-fiber nociceptors [53]. In a PHN rat model, inducible nitric oxide synthase (iNOS) in astrocytes, which produce large amounts of NO, is induced in response to VZV infection [54]. NO activates NMDA receptors in spinal dorsal horn neurons through phosphorylation, resulting in mechanical allodynia [10]. Therefore, the experimental model demonstrated VZV-NO-astrocyte-induced allodynia. Besides, vitamin D is anti-neuroinflammatory [6,7,52]. During inflammation, activated glia and astrocytes may synthesize 1,25(OH)D which inhibits iNOS expression and reduces the production of NO [8,9]. In mice, vitamin D deficiency generates reduced mechanical threshold without altering the thermal nociceptive threshold [16]. Vitamin D deficiency also induces a significant increase in the spontaneous activity and activation frequency of spinal nociceptive specific neurons as well as the duration of the evoked activity of spinal nociceptive specific neurons [16]. Accordingly, symptoms and signs in PHN patients may be augmented by hypovitaminosis D, perpetuating a vicious cycle involving spontaneous pain/allodynia and hypovitaminosis D at the molecular level. As for malignancy [55] and *Helicobacter pylori*-related peptic ulcer disease [38], both have been identified as the risk factors of PHN based on previous reports.

In Part II study, hypovitaminosis D was found to be associated with increased neuropathic pain in PHN patients. Vitamin D is a neuroactive steroid that may mediate pain processes by modulating several signal transduction systems. Patients with statin-induced musculoskeletal pain often have low vitamin D levels [56]. Statins decrease cholesterol synthesis through the reversible block of the hydroxy-3-methylglutaryl-coenzyme A reductase. As a result, statins may reduce the production of 7-dehydrocholesterol which can be photochemically converted to pre-vitamin D in the skin. Subsequently, pre-vitamin D is metabolized into 25(OH)D in the liver and is then converted into 1,25(OH)₂D in the kidney. Vitamin D is essential for the maintenance of musculoskeletal health; thus, its deficiency may produce muscular weakness and pain. A recent study showed a significant negative correlation between vitamin D levels and the severity of pain in patients with lower back pain [57]. Seemingly, patients suffering from chronic pain often have hypovitaminosis D. Recently, Guida et al. [16] demonstrated that spared nerve injury in normal or vitamin D deficient mice does not induce changes in gut microbiota. Nonetheless, vitamin D deficiency induces a marked dysbiosis (i.e., a lower microbial diversity characterized by an increase in Firmicutes and a decrease in Verrucomicrobia and Bacteroidetes). In addition, vitamin D deficiency alters the endocannabinoid system through reducing the expression of spinal cannabinoid receptor type 1, increasing levels of spinal cannabinoid receptor type 2 as well as changing endocannabinoid and endocannabinoid-like mediator levels in the gut. Concurrently, vitamin D deficiency causes tactile allodynia associated with spinal neuronal sensitization. Vitamin D deficiency affects nociception possibly via molecular mechanisms involving the endocannabinoid and related mediator signaling systems. Based on the findings of our studies and those of others, screening for hypovitaminosis D is suggested in the management of PHN.

In the current study, the cutoffs for 25(OH)D level to predict spontaneous pain and allodyniawere67.0 and 169.0 nmol/L, respectively. The difference in thresholds is consistent with the finding of a previous experimental study showing different thresholds for eliciting pain, allodynia, and hyperalgesia in rats [58]. In 88 PHN patients, 74 (84.1%) had a score greater or equal to 4 in the DN4 questionnaire. Our results suggest that a physical examination by the physician is still necessary [29]. Compared to patients with vitamin D >67.0 nmol/L, those with vitamin D ≤ 67.0 nmol/L had significantly greater proportions of painful cold among the seven symptoms in the DN4 questionnaire. The results support those of previous reports showing that lower 25(OH)D levels were correlated to lower cold detection thresholds in patients with painful diabetic peripheral neuropathy [19]. However, our findings demonstrated no association between 25(OH)D level and burning pain or other symptoms in PHN. In mice, vitamin D deficiency increases the production of ROS [11] which activates TRPA1 [12] and TRPV1 [59]. Sensitization of TRPA1 via ROS signaling causes noxious cold pain [13]. On the other hand, vitamin D inhibits TRPV1 channels which are involved in thermal hyperalgesia only in the acute phase of neuropathic pain [59]. The results of animal models support our clinical findings. Importantly, vitamin D supplementation has been demonstrated to reduce neuropathic pain in vitamin D-insufficient diabetic patients [18,60]. Optimization of vitamin D status may potentially prevent and treat PHN as an alternative therapy for spontaneous pain (painful cold) and allodynia.

The positive rate of VZV IgM in shingles ranges from 10% to 70% [45]. The positive rate of VZV IgM in our PHN patients was 11.4%. The current study is the first to report the positive rate of VZV IgM in patients with PHN. Our results showed that VZV IgM titer was positively correlated to spontaneous pain. Our findings support that some PHN is associated with VZV ganglionitis caused by persistent viral infection [48] and that high VZV IgM titers in zoster patients imply a high risk for PHN [61,62]. The cutoff for IgM to predict spontaneous pain was 0.60, indicating that antiviral therapy may decrease pain for patients with IgM ≥ 0.60. However, the analgesic efficacy of antiviral therapy for PHN remains conflicting [48,63,64]. This may be attributed to the lack of information on the concentrations of VZV immunoglobulins in those clinical trials. Further studies are needed to elucidate whether antiviral therapy is more effective in patients with high IgM titers than those with low titers as well as to assess the impact of hypovitaminosis D on the efficacy of antiviral therapy for PHN [35,65].

In terms of limitations, we did not assess sun exposure time, sunscreen use, daily activity, and vitamin D-rich food consumption [3], although the effects of these factors on our findings are likely to be limited. Second, patients aged >85 years were not included due to a lack of age-matched controls. Third, although race is a risk factor of hypovitaminosiss D [66], only Taiwanese were enrolled in this study. Further studies on other ethnic groups are warranted to generalize the results. Fourth, because of a small patient number and the retrospective nature of the present study, it is impossible to draw conclusions about causality. This demonstrates the need for large-scale prospective cohort studies to identify the causes and underlying mechanisms of hypovitaminosis D in PHN. 

## 5. Conclusions

PHN patients aged 50–85 had a high prevalence of hypovitaminosis D which was associated with increased spontaneous and brush-evoked pain. PHN patients with hypovitaminosis D had greater pain, higher VZV IgM titers, and a lower vitamin D supplementation rate than those in subjects without the condition. The cutoffs for 25(OH)D to predict spontaneous pain and brush-evoked pain were 67.0 and 169.0 nmol/L, respectively. Patients with vitamin D ≤ 67.0 nmol/L had significantly greater proportions of painful cold. Spontaneous pain was also correlated to VZV Ig Mtiters. The cut-off for IgM to predict spontaneous pain was 0.60. Further prospective, longitudinal studies are warranted to confirm these findings.

## Figures and Tables

**Table 1:** Conditional logistic regression analysis of potential predictors for PHN.

Predictors	PHN (*n* = 88)	Controls (*n* = 264)	Univariate OR (95% CI)	*p*	Adjusted OR (95% CI)	*p*
Age, years, mean (SD)	65.3 (9.4)	65.3 (9.0)		0.997		
Age groups						
≥60 years	64 (72.7%)	192 (72.7%)				
50–59 years	24 (27.3%)	72 (27.3%)				
Gender						
Male, *n* (%)	47 (53.4%)	141 (53.4%)				
Body mass index, mean (SD)	23.68 (3.26)	23.99 (3.07)		0.426		
Body mass index (kg/m^2^)			1.29 (0.54-3.06)	0.563	1.01 (0.36-2.79)	0.990
<18.5 or ≥30	8 (9.1%)	19 (7.2%)				
18.5~30	80 (90.0%)	245 (92.8%)				
25(OH)D (nmol/L), mean (SD)	68.96(18.72)	75.13 (17.47)		0.005		
Vitamin D status			3.31 (1.92-5.72)	<0.001	3.12 (1.73-5.61)	<0.001 *
Sufficiency, *n* (%)	23 (26.1%)	140 (51.9%)				
Hypovitaminosis D, *n* (%)	65 (73.9%)	124 (47.0%)				
Comorbidities						
Hypertension	33 (37.5%)	84 (31.8%)	1.35 (0.78-2.37)	0.279	1.14 (0.59-2.17)	0.702
Diabetes mellitus	26 (29.5%)	42 (15.9%)	2.22 (1.26-3.90)	0.005	1.97 (0.96-4.06)	0.065
Malignancy	15 (17.0%)	18 (6.8%)	2.71 (1.31-5.59)	0.007	3.21 (1.38-7.48)	0.007 *
Chronic liver disease	10 (11.4%)	28 (10.6%)	1.08 (0.51-2.28)	0.846	1.24 (0.52-2.93)	0.630
Chronic kidney disease	2 (2.3%)	6 (2.3%)	1.00 (0.20-4.95)	1.000	0.75 (0.13-4.48)	0.757
Autoimmune diseases	8 (9.1%)	10 (3.8%)	2.40 (0.95-6.08)	0.065	2.85 (0.98-8.27)	0.055
H. pylori-related PUD	23 (26.1%)	25 (9.5%)	3.15 (1.70-5.84)	<0.001	3.47 (1.71-7.03)	0.001 *
Antiviral therapy	38 (43.2%)	-				
Average spontaneous pain, mean (SD) (NRS 0–10)	5.84 (1.46)	-				
Brush-evoked pain, mean (SD) (NRS 0–10)	3.14 (3.10)	-				

*n*: number; SD: standard deviation; PHN: postherpetic neuralgia; 25(OH)D: serum 25-hydroxyvitamin D; PUD: peptic ulcer disease; NRS: numeric rating pain scale. T-test was used for continuous data. Chi Square or Fisher exact test was used for categorical data. Adjusted OR was determined using the conditional multiple logistic regression model by gender, age and index season match. * A *p*-value <0.05 was considered significant. Chronic liver disease: Patients had chronic hepatitis B and/or C or liver cirrhosis. Chronic kidney disease: Patients had hemodialysis. *Helicobacter pylori*-related PUD was defined as either positive findings on hospital gastroduodenoscopy records or a self-reported gastroduodenoscopy history with prescriptions for peptic ulcers/gastritis within one year prior to a shingles outbreak. -: The controls did not receive any antiviral therapy for VZV or pain measurement.

**Table 2:** Demographic and clinical characteristics of patients with hypovitaminosis D vs. sufficiency of vitamin D.

	Hypovitaminosis D (*n* = 65)	Sufficiency of vitamin D (*n* = 23)	*p*
Age group			0.075
≥60 years, *n* (%)	44 (67.7)	20 (87.0)	
Gender			0.071
Male, *n* (%)	31 (47.7)	16 (69.6)	
Body mass index (kg/m^2^)			0.357
<18.5 or ≥30, *n* (%)	7 (10.8)	1 (4.3)	
VZV-IgG (mIU/mL), mean (SD)	4239 (1382)	4281 (1066)	0.955
VZV-IgG, positive, *n* (%)	65 (100)	23 (100)	1.0
VZV-IgM, mean (SD)	0.63 (0.45)	0.40 (0.25)	0.016 *
VZV-IgM, positive, *n* (%)	8 (12.3)	1 (4.3)	0.279
Comorbidities, *n* (%)			
Hypertension	25 (38.5)	8 (34.8)	0.754
Diabetes mellitus	21 (32.3)	5 (21.7)	0.340
Malignancy	12 (18.5)	3 (13.0)	0.553
Chronic liver disease	8 (12.3)	2 (8.7)	0.639
Chronic kidney disease	2 (3.1)	0 (0.0)	0.416
Autoimmune diseases	6 (9.2)	2 (8.7)	0.939
*Helicobacter pylori*-related PUD	19 (29.2)	4 (17.4)	0.267
Vitamin D supplements★, *n* (%)	1 (1.5)	4 (17.4)	0.005 *
Average spontaneous pain, mean (SD) (NRS 0–10)	6.1 (2.1)	5.3 (1.8)	0.021 *
Brush-evoked pain, mean (SD) (NRS 0–10)	4.3 (6.8)	2.5 (8.3)	0.007 *

*n*: number; VZV: varicella-zoster virus; PUD: peptic ulcer disease; NRS: numeric rating pain scale. VZV-IgG, positive: >110 mIU/mL; VZV-IgM, positive: ≥1.0. T-test was used for continuous data. Chi Square or Fisher exact test was used for categorical data. ★ All of the five patients irregularly received a self-prescribed supplement of vitamin D (400 or 800 IU/day). * A *p*-value <0.05 was considered significant.

**Table 3:** Correlations between NRS of pain and serum concentrations of 25(OH)D/VZV Igs in PHN.

Correlation	Spearman’s Correlation Coefficient	*p*
Spontaneous pain (NRS 0-10) vs.		
brush-evoked pain (NRS 0–10)	0.196	0.067
25(OH)D (nmol/L)	−0.329 *	0.002
VZV IgG(mIU/ml)	0.249	0.019
VZV IgM	0.363 *	0.001
Brush-evoked pain (NRS 0-10) vs.		
25(OH)D (nmol/L)	−0.311 *	0.003
VZV IgG(mIU/ml)	−0.181	0.092
VZV IgM	−0.183	0.088

Ig: Immunoglobulin; NRS: 11-point numeric rating pain scale (0–10); 25(OH)D: 25-hydroxyvitamin D; VZV: varicella-zoster virus; PHN: postherpetic neuralgia. * Spearman correlation coefficients indicate clinical significance if the value is greater than 0.3.

**Table 4:** Proportions of items in the DN4 questionnaire between patients with serum 25(OH)D concentration >the cutoff value vs.≤ the cutoff value.

Cutoff	25(OH)D		*p*	Insufficiency Deficiency		*p*
	>67.0 nmol/L (*n* = 46)	≤ 67.0nmol/L (*n* = 42)		50–75nmol/L (*n* = 55)	<50.0 nmol/L (*n* = 10)	
Burning pain, *n* (%)	28 (56.0)	22 (44.0)	0.422	14 (25.5)	2 (20.0)	0.713
Painful cold, *n* (%)	2 (11.8)	15 (88.2)	<0.001 *	10 (18.2)	6 (60.0)	0.005 *
Electric sharp pain, *n* (%)	35 (53.8)	30 (46.2)	0.619	9 (16.4)	2 (20.0)	0.778
Tingling, *n* (%)	35 (47.9)	38 (52.1)	0.073	33 (60.0)	7 (70.0)	0.550
Pins and needles, *n* (%)	36 (49.3)	37 (50.7)	0.220	31 (56.4)	6 (60.0)	0.831
Numbness, *n* (%)	19 (54.3)	16 (45.7)	0.759	17 (30.9)	4 (40.0)	0.572
Itching, *n* (%)	15 (32.6)	13 (31.0)	0.868	17 (30.9)	2 (20.0)	0.485
Hypoesthesia to touch, *n* (%)	17 (50.0)	17 (50.0)	0.735	21 (38.2)	4 (40.0)	0.913
Hypoesthesia to pinprick, *n* (%)	13 (46.4)	15 (53.6)	0.453	19 (34.5)	3 (30.0)	0.780
Brush-evoked pain, *n* (%)	27 (42.2)	37 (57.8)	0.002 *	43 (78.2)	10 (100.0)	0.225
DN4 ≥4, *n* (%)	39 (84.8)	35 (83.3)	0.853	45(81.8)	9 (90.0)	0.526

*n*: number; VZV: varicella-zoster virus; DN4: the Douleur Neuropathique 4 questionnaire. * A *p*-value <0.05 was considered significant.

**Table 5:** Proportions of items in the DN4 questionnaire between patients with VZV IgM titer ≥the cutoff value vs. < the cutoff value.

Cutoff	VZV IgM		*p*
	≥0.6 (*n* = 37)	<0.6 (*n* = 51)	
Burning pain, *n* (%)	19 (38.0)	31 (62.0)	0.378
Painful cold, *n* (%)	7 (41.2)	10 (58.8)	0.936
Electric sharp pain, *n* (%)	27 (41.5)	38 (58.5)	0.871
Tingling, *n* (%)	30 (41.1)	43 (58.9)	0.691
Pins and needles, *n* (%)	32 (43.8)	41 (56.2)	0.453
Numbness, *n* (%)	14 (40.0)	21 (60.0)	0.752
Itching, *n* (%)	12 (32.4)	16 (31.4)	0.916
Hypoesthesia to touch, *n* (%)	13 (38.2)	21 (61.8)	0.566
Hypoesthesia to pinprick, *n* (%)	12 (42.9)	16 (57.1)	0.916
Brush-evoked pain, *n* (%)	28 (43.8)	36 (56.3)	0.597
DN4 ≥4, *n* (%)	30 (81.1)	44 (86.3)	0.511

*n*: number; VZV: varicella-zoster virus; DN 4: the Douleur Neuropathique 4 questionnaire.

## Data Availability

Anonymized data not published within this article will be made available and shared by request from any qualified investigator.

## Supplementary Materials

The following are available online at https://www.mdpi.com/2072-6643/11/11/2787/s1, Supplementary S1. First-visit questionnaire to the pain clinic at Chi Mei Medical Center.

## Abbreviations

25(OH)D	25-hydroxyvitamin D
AUC	area under the ROC curve
DN4	Douleur Neuropathique 4
ELISA	Enzyme Linked Immunosorbent Assay
ICD-9	International Classification of Diseases, Ninth Revision, Clinical Modification
iNOS	inducible nitric oxide synthase
NMDA	N-methyl-D-aspartate
NO	nitric oxide
NRS	numeric rating pain scale
PHN	postherpetic neuralgia
ROC curve	receiver operating characteristic curve
ROS	reactive oxygen species
TLR	toll-like receptor
TRPA1	transient receptor potential ankyrin 1
VZV	varicella-zoster virus
