# Supplementary material for: Hypovitaminosis D in Postherpetic Neuralgia—High Prevalence and Inverse Association with Pain: A Retrospective Study
Source: Nutrients. 2019 Nov 15;11(11):2787. doi: 10.3390/nu11112787 (PMC6893816; doi:10.3390/nu11112787)
Supplement: Supplementary file 1 [file nutrients-11-02787-s001.pdf]

Supplementary S1. **First-visit questionnaire to the pain clinic at Chi Mei Medical Center**
First visit date: (dd/mm/yy)
Name: ; Medical record number: ; Birthday:

(1) Basic information:
Diet: vegetarian: No/Yes, (1. breakfast only; 2. two days a month; 3. always)
Smoking: No/Yes, years; package/day; quitted for years
Drinking: No/Yes, year; (Frequency: every day, days a week; bottles/time; totally mL; Beer/sorghum/Whiskey/)
Betel nut: No/Yes, for years; pack/day; quitted for years

(2) Past medical history:
Hypertension: No/Yes, for years (Medication:) BP: HR:
Coronary heart disease: No/Yes, for years (Medication: ; stenting: years ago)
Stroke: No/Yes (times, years ago)
Diabetes: No/Yes, for years (Medication: /RI year).
Cancer: No/Yes. (year ago)- left/right (breast/lung/kidney) / pancreas / colon /
Treatment: Surgery/Chemotherapy/Radiotherapy/Others:
Autoimmune diseases (SLE, RA, Sjogren syndrome,): No/Yes, for years
Hepatitis B/C carrier; liver cirrhosis: No/Yes;
Chronic kidney disease, hemodialysis/PD: No/Yes, for years (weekdays 1,3,5/2,4,6)
Peptic ulcers: No/Yes, for years (post treatment- years ago)
Helicobacter pylori: No/Yes, for years
Other diseases: COPD: No/Yes.; Hyperuricemia: No/Yes.; Glucose-6-phosphate dehydrogenase (G6PD) deficiency: No/Yes.;
Surgery history (site / time / hospital):

Special medications: steroid, drug abuse,
Drug allergy: No/Yes, (Drug: ; Symptoms: rash / /shock)
Supplementation : No/Yes, Regular/Irregular (multivitamins ; calcium mg/day; vitamin C mg/day; vitamin D iu/day; fish oil mg/day)

(3) Family history
Father: Diabetes / Hypertension / Stroke / Tumor
Mother: Diabetes / Hypertension / Stroke / Tumor;

(4) History of herpes zoster
Sites: face/neck and shoulder/upper extremity/trunk/sacral/lower extremity
Nerve roots: left/right; C/T/L/S ________________________
Date of shingles outbreak:__________________________
Duration (from the zoster onset to the first visit at our pain clinic): ______ weeks/months
Referral to our pain clinic: due to (A); (B); (C) both _______
(A) Poor response to treatment
(B) Intolerableside effects (dizziness / nausea and vomiting / constipation / hand and foot swelling /)
Have you taken antiviral drugs? No/Yes, for days. /Don't know.
Have you taken lyrica /neurontin / morphine? No/Yes, dosage:
Have you received nerve blocks ( )?

(5) DN4 Questionnaire - ___ /10
QUESTION 1:

Does the pain have one or more of the following characteristics? YES NO
(5-1) Burning . . . . ❑ ❑
(5-2) Painful cold . . . . ❑ ❑
(5-3) Electric shocks . . . . ❑ ❑
QUESTION 2:
Is the pain associated with one or more of the following symptoms in the same area?
YES NO
(5-4) Tingling . . . . ❑ ❑
(5-5) Pins and needles . . . . ❑ ❑
(5-6) Numbness . . . . ❑ ❑
(5-7) Itching . . . .❑ ❑
QUESTION 3:
Is the pain located in an area where the physical examination may reveal one or more of the following characteristics? YES NO
(5-8) Hypoesthesia to touch . . . . ❑ ❑
(5-9) Hypoesthesia to pinprick . . . .❑ ❑
QUESTION 4:
(5-10) In pain area, can the pain be caused or increased by Brushing?YES NO
. . . .❑ ❑

(6-1) NRS scales (0-10) of average spontaneous pain:
(6-2) NRS scales (0-10) of the worst spontaneous pain:
(6-3) NRS scales (0-10) of brush-evoked pain:

	**YES**	**NO**
(7) Other characteristics of pain: spasticity	❑	❑
swelling pain	❑	❑
aching pain	❑	❑
dull pain	❑	❑
sharp pain	❑	❑

more pain when the weather temperature increased

more pain when the weather temperature dropped
